# In Situ Deposition of Gold Nanoparticles and L-Cysteine on Screen-Printed Carbon Electrode for Rapid Electrochemical Determination of As(III) in Water and Tea

**DOI:** 10.3390/bios13010130

**Published:** 2023-01-12

**Authors:** Wenjing Wang, Zhijian Yi, Qiongxin Liang, Junjie Zhen, Rui Wang, Mei Li, Lingwen Zeng, Yongfang Li

**Affiliations:** 1 School of Food Science and Engineering, Foshan University, Foshan 528231, China; 2 Guangdong Langyuan Biotechnology Co., Ltd., Foshan 528313, China; 3 State Key Laboratory of Genetic Engineering, Human Phenome Institute, Fudan University, Shanghai 200438, China; 4 Wuhan Zhongkezhikang Biotechnology Co., Ltd., Wuhan 430223, China

**Keywords:** As(III), L-cysteine, in situ deposition, screen-printed carbon electrodes, linear sweep anodic stripping voltammetry

## Abstract

In this study, a screen-printed carbon electrode (SPCE) based on in situ deposition modification was developed for the sensitive, rapid, easy and convenient determination of As(III) in water and tea by linear sweep anodic stripping voltammetry (LSASV). The screen-printed carbon electrodes were placed in a solution consisting of As(III) solution, chlorauric acid and L-cysteine. Under certain electrical potential, the chloroauric acid was reduced to gold nanoparticles (AuNPs) on the SPCE. L-cysteine was self-assembled onto AuNPs and promoted the enrichment of As(III), thus enhancing the determination specificity and sensitivity of As(III). The method achieved a limit of determination (LOD) of 0.91 ppb (µg L^−1^), a linear range of 1~200 µg L^−1^, an inter-assay coefficient of variation of 5.3% and good specificity. The developed method was successfully applied to the determination of As(III) in tap water and tea samples, with a recovery rate of 93.8%~105.4%, and further validated by inductively coupled plasma mass spectrometry (ICP-MS). The developed method is rapid, convenient and accurate, holding great promise in the on-site determination of As(III) in tap water and tea leaves, and it can be extended to the detection of other samples.

## 1. Introduction

In recent years, heavy metal pollution has been intensified with the increase in industrial activities, making drinking water and food vulnerable to contamination [1,2,3]. Heavy metal ion contaminants are not easily degraded. They can enrich and accumulate in the human body through the food chain, and even trace amounts of heavy metals can cause damage to people’s health and safety [4,5]. Among them, metalloid arsenic (As) contamination is one of the most widespread, toxic and harmful problems [6,7]. The World Health Organization (WHO) has set the maximum allowable concentrations for As to be 10 ppb (10 μg L^−1^, 133 nM) in drinking water [8]. Therefore, the rapid, sensitive and accurate determination of As in water, food and the environment is essential.

Traditional methods for As determination focused on atomic absorption spectrometry (AAS), atomic fluorescence spectrometry (AFS) and inductively coupled plasma mass spectrometry (ICP-MS) [9,10,11,12]. Although these methods have the advantages of high sensitivity, accuracy and stability, their reliance on bulky and expensive instruments makes it difficult to realize rapid detection for field applications. Electrochemical methods, which use a variety of nano and micron materials to fabricate sensors, exhibit advantages of good selectivity, high sensitivity, a fast response and a low cost [13]. Combined with portable detection equipment, electrochemical methods offer great promise for field detection [14]. Anodic stripping voltammetry (ASV) is a sensitive and efficient electrochemical method for heavy metal ion detection [15]. Linear sweep anodic stripping voltammetry (LSASV) is one of the most commonly used electrochemical methods. It can be well applied to small portable devices due to the small sample volume required [14]. LSASV detects arsenic based on the following principles: a reduction reaction is first carried out to reduce As^3+^ to As^0^, which is deposited on the electrode surface; an oxidation reaction is then carried out to oxidize the As^0^ deposited on the surface of the electrode to As^3+^, which is stripped out into the solution matrix [16]. Each metal ion has its own specific oxidation potential and can therefore be well differentiated. The potential at which the stripping of a metal ion occurs is the characteristic potential of this metal ion. The current that it generates is proportional to the concentration of the metal ion in solution. Screen-printed carbon electrodes (SPCEs) are widely used in electrochemical detection due to their low cost, good repeatability and mass production. An SPCE modified with nanomaterials is an effective and promising method for the sensitive and rapid detection of heavy metal ions.

Much effort has been devoted to the modification of SPCEs for the determination of As(III) [17,18,19,20,21,22]—for example, SPCEs functionalized with aptamers for the detection of total arsenic in shellfish [23]; gold-nanoparticle-modified SPCEs for the determination of As(III) [24]; SPCEs modified with silica nanoparticles [25]; gold nanoparticles with fecal-*Bacillus*-modified SPCEs for the determination of As(III) [26]; and self-assembled single molecular layer electrochemical methods for the determination of As(III) [27]. Although these methods have good sensitivity, they usually synthesize nanomaterials primarily and then coat them onto the surface of the SPCE by dropping, which may cause a “coffee ring effect” and affect the detection performance [28,29]. In addition, gold nanoparticles fabricated on an SPCE may degrade quickly at room temperature [30]. Moreover, these methods require the fabrication of the sensor before detection, which is not suitable for rapid detection in the field. For the better determination of arsenic, a new, fast, simple, sensitive and environmentally friendly method is urgently needed.

**Figure 1:**
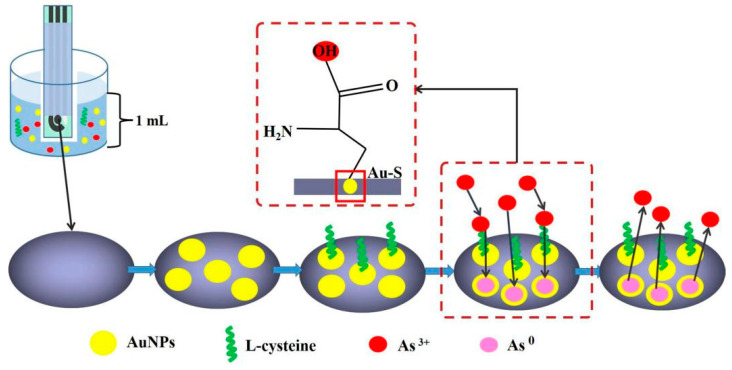
The in situ deposition of gold nanoparticles and L-cysteine on SPCE for As(III) determination.

Herein, we developed an SPCE for As(III) determination based on the in situ deposition of gold nanoparticles and L-cysteine. The SPCE was placed in a 1 mL sample cell consisting of As(III) solution, chloroauric acid and L-cysteine. Chloroauric acid is reduced to gold nanoparticles on the SPCE when a certain voltage is applied. The sulfur bond at one end of L-cysteine recognizes the gold nanoparticles on the SPCE to form a stable complex through the Au-S bond. The −OH at the other end of L-cysteine has weak adsorption for As(III), which can help the adsorption of As on gold nanoparticles. Moreover, L-cysteine can mask the interference of some metal ions. When a negative electrical potential is applied, arsenic ions in solution are reduced and enriched onto the surface of the working electrode of the SPCE; the arsenic is then oxidized and stripped off the SPCE when a positive electrical potential is applied (Figure 1). The stripping current is the signal for the determination of arsenic. Linear sweep anodic stripping voltammetry (LSASV) was applied for the determination of As(III). The limit of determination (LOD) is 0.91 μg L^−1^, the whole test takes around 24 min, and the detection cost is around USD 0.14. In addition, the method was verified using tap water and tea leaf samples and the measured results were consistent with the ICP-MS results.

## 2. Experimental Section

### 2.1. Reagents and Chemicals

Chloroauric acid tetra hydrate, L-cysteine, K_3_[Fe (CN)_6_] and K_4_[Fe (CN)_6_] were purchased from Shanghai Macklin Biochemical Technology Co. Sulfuric acid was purchased from Guangzhou Chemical Reagent Factory. As(III) and other metal ion solutions were prepared from 1000 mg L^−1^ stock solutions (Merck, Darmstadt, Germany). Electrolyte solution was prepared as 50 µM chloroauric acid and 0.4 mM L-cysteine in 50 mM H_2_SO_4_. As(III) stock solution was serially diluted with 0.1 M H_2_SO_4_ to obtain a series of As(III) standard solutions. All solutions were prepared with ultrapure water produced with Milli-Q (Millipore, Burlington, MA, USA).

### 2.2. Apparatus

The electrochemical measurements were carried out with a CHI1440 constant potential instrument (Shanghai CH Instruments Co., Ltd., Shanghai, China). Scanning electron microscopy (SEM) images were obtained on a cold field emission scanning electron microscope (ULTRA55 ZEISS, Ltd., Oberkochen, Germany). All electrochemical experiments were performed on a disposable SPCE, which consisted of a carbon working electrode with a diameter of 2.8 mm, a carbon counter electrode and an Ag/AgCl reference electrode (Guangdong Langyuan Biotechnology Co., Ltd., Foshan, China). An SPCE connector and 1 mL plastic sample cell were mounted on a self-made, time- and speed-regulated stirring device (Figure S1), which was used to rotate the sample cell and provide stirring during the enrichment step in the anodic stripping voltammetry. The home-made device is a good substitute for the traditional magnetic stirrer, which allows the use of a low sample volume (1 mL) and miniaturization of the assay instrument.

### 2.3. Methods

For electrochemical measurement, an SPCE was immersed in a 1 mL sample cell with 800 µL of electrolyte solution and 200 µL of the As(III) standard solution. The electrochemical method used was LSASV. The LSASV detection parameters were as follows. The scan window was from −0.15 V to 0.5 V; the scan rate was 2 V/s; the sampling interval was 0.001 V; the rest time was 10 s; the deposition time was 1400 s; and the deposition potential was −0.8 V. The stirring speed during deposition was set as 100 rpm, and no stirring was provided during the stripping process. All assays were carried out at room temperature.

### 2.4. Recovery Studies

Tap water and 3 brands of tea samples without arsenic were used for recovery studies. The tea samples were crushed into powders. One microliter of arsenic solution containing 1000 µg L^−1^ of arsenic was spiked into 20 mL of tap water and 0.5 g of tea powder, respectively. The spiked tap water samples were filtered through a 0.22 µm microporous membrane. The spiked tea powder samples were dissolved in 0.1 M H_2_SO_4_, and then boiled for 15 min. After cooling to room temperature, the tea solution was allowed to stand for 5 min. The supernatant was filtered through a 0.22 µm microporous membrane. Voltammetry was carried out on 200 µL of filtered tap water and filtered tea samples using the method described in Section 2.3. The spiked samples were also analyzed using inductively coupled plasma mass spectrometry (ICP-MS).

## 3. Results and Discussion

### 3.1. Morphological Characterization of SPCEs

**Figure 2:**
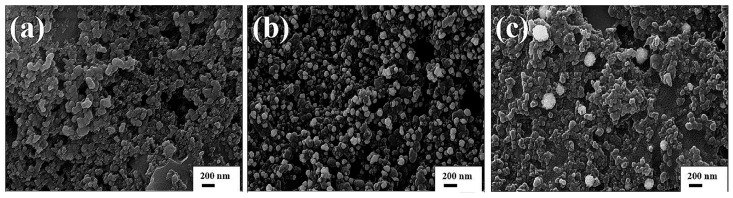
The SEM images of the SPCE showing (**a**) bare SPCE, (**b**) AuNP-modified SPCE and (**c**) AuNP/L-cysteine-modified SPCE.

The surface morphology of the three different SPCEs was characterized by SEM. Figure 2a,b show a dramatic difference in the electrode surface before and after the deposition of gold nanoparticles. The AuNPs with an average size of approximately 100 nm are deposited to form a rough surface, whereas no such deposition is observed for the bare electrode and the surface is very smooth. Furthermore, the surface morphology of the electrode modified with AuNPs and L-cysteine is significantly different from that of the AuNP-modified electrode without L-cysteine (Figure 2b,c). With the addition of L-cysteine, the AuNPs aggregated to form AuNP clusters. The diameters of the AuNP clusters are around 200 nm, and the edges of the AuNP clusters are defocused (Figure 2c). A plausible explanation is that in the presence of L-cysteine, the growth of AuNPs was retarded during deposition due to the binding of L-cysteine to the AuNPs through Au-S bonds [31,32].

### 3.2. Electrochemical Characterization of Electrodes

#### 3.2.1. Cyclic Voltammetry Characterization of Gold Nanoparticles on SPCEs

**Figure 3:**
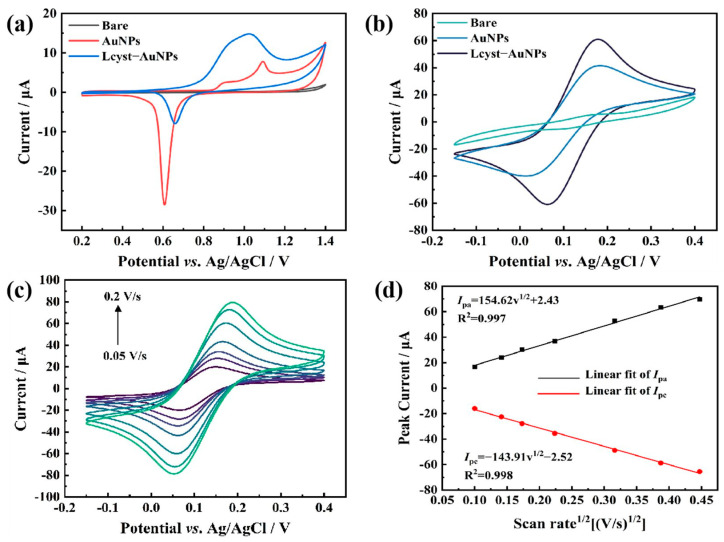
Electrochemical characterization of electrodes. (**a**) Cyclic voltammograms for bare SPCE, AuNP-modified SPCE and AuNP/L-cysteine-modified SPCE at 0.5 mol L^−1^ H_2_SO_4_. (**b**) Cyclic voltammograms for bare SPCE, AuNP-modified SPCE and AuNP/L-cysteine-modified SPCE in 5 mmol L^−1^ [Fe (CN)_6_]^3−/4−^ + 0.1 mol L^−1^ KCl solution. (**c**) Cyclic voltammograms of gold nanoparticle and L-cysteine co-deposited SPCE at different scan rates. (**d**) Linear plotting of peak current values versus the square root of the scan rate.

The modified SPCEs were subjected to cyclic voltammetry (CV) scans using 0.5 mol L^−1^ H_2_SO_4_. The scan range was from 0.2 V to 1.4 V and the scan rate was 0.1 V/s. The scan results are shown in Figure 3a. The gold-nanoparticle-modified electrode showed a characteristic reduction peak of AuNPs that appeared at 0.62 V, indicating that the chloroauric acid was reduced to AuNPs [33]. When L-cysteine was added for in situ co-deposition with chloroauric acid, the characteristic reduction peak height of AuNPs decreased significantly, while the oxidation peak at 1.05 V increased and broadened. The decreased reduction peak of AuNPs can be explained by the fact that the L-cysteine absorbed on the AuNPs can reduce the growth rate of AuNPs. Meanwhile, the binding of L-cysteine on AuNPs does not affect the oxidation of the AuNPs as L-cysteine falls away from the AuNPs during the oxidation process. In addition, the AuNP clusters formed on the AuNP and L-cysteine co-deposited electrode significantly increased the surface area of the electrode, therefore increasing the oxidation current peak [34].

#### 3.2.2. Electrode Performance Evaluation with Cyclic Voltammetry

Bare electrode and electrodes with different modifications were evaluated with cyclic voltammetry. The CV scan was carried out in 5 mmol L^−1^ [Fe (CN)_6_]^3−/4−^ and 0.1 mol L^−1^ KCl solution. The scan range was from −0.15 V to 0.4 V and the scan rate was 0.1 V/s. The results are shown in Figure 3b. The redox current of the AuNP-modified electrode was much higher than that of the bare electrode. The higher redox current indicates a higher electron transfer rate and better conductivity. The rough surface of the AuNP-deposited electrode increased the effective area of the electrode [35], therefore improving the performance of the electrode. The redox current increased further for the gold nanoparticle and L-cysteine co-deposited SPCE (Figure 3b). It is obvious that the addition of L-cysteine did not block the electron transfer during the redox reaction. On the contrary, it enhanced the conductivity of the electrode. The redox peak potential difference of the AuNP-modified electrode without L-cysteine was 0.16 V. The redox peak potential difference of the AuNP-and-L-cysteine-modified electrode was 0.11 V. The gold nanoparticle and L-cysteine co-deposited SPCE showed a smaller redox peak potential difference and therefore a greater degree of reversibility in comparison with the electrodes modified with AuNPs without L-cysteine (Figure 3b). The clusters of AuNPs formed in the presence of L-cysteine increased the effective surface area further and therefore contributed to the better performance of the gold nanoparticle and L-cysteine co-deposited SPCE.

#### 3.2.3. Measurement of Effective Surface Area of Electrodes

The effective areas of the bare electrodes and modified electrode were measured. Figure 3c shows the CV curves of different electrodes in 0.1 mol L^−1^ KCL and 5 mmol L^−1^ [Fe (CN)_6_]^3−/4−^ solution at different scan rates. The scan range was −0.2~0.4 V and the scan rate varied from 0.05 V/s to 0.2 V/s. The peak current values were in a good linear relationship with the square root of the scan rate (Figure 3d), indicating that the oxidation–reduction process of [Fe (CN)_6_]^3−/4−^ on the modified electrode was mainly based on linear diffusion. Therefore, the effective area was estimated by the Randles–Sevcik equation [36,37]:

Ip = 2.69 × 10^5^n^3/2^*AD*^1/2^υ^1/2^*C*_0_

Ip is the oxidation peak current, n is the number of transferred electrons (n = 1), *A* is the effective area of the working electrode (cm^2^), *D* is the diffusion coefficient of the substance (*D* = 6.30 × 10^−6^ cm^2^ s^−1^), *C*_0_ is the concentration of the substance (mol cm^−3^) and υ is the scan rate (V s^−1^). It is calculated that the effective area of the bare electrode is 0.002 cm^2^, the effective area of the AuNP-modified electrode is 0.029 cm^2^, and the effective area of the gold-nanoparticle-and-L-cysteine-modified electrode is 0.049 cm^2^. The effective area of the modified electrode is much larger than that of the bare electrode. The increased effective surface area explains the improved electrochemical performance of the modified electrode.

### 3.3. Electrochemical Behavior of Arsenic on Different Electrodes

**Figure 4:**
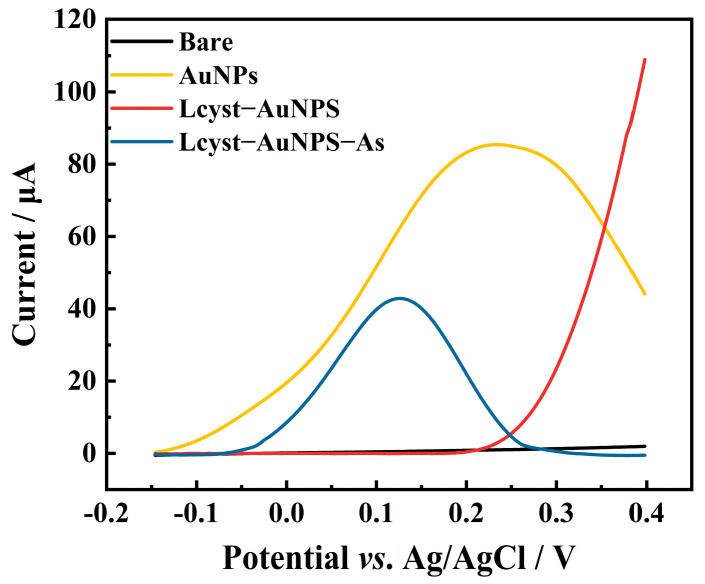
LSASV curves for the determination of arsenic on different SPCEs (800 µL electrolyte solution: 50 µM chloroauric acid and 0.4 mM L-cysteine in 50 mM H_2_SO_4_; 200 µL As(III) standard solution: 50 µg L^−1^. LSASV: scan window −0.15 V to 0.5 V; scan rate is 2 V/s; sampling interval is 0.001 V; rest time is 10 s; deposition time is 1400 s; deposition potential is −0.8 V; stirring speed during deposition was set as 100 rpm).

Three different electrodes were used to detect As(III) using LSASV in a solution containing 50 μg L^−1^ of As(III). As shown in Figure 4, no stripping peak of As was observed for the bare electrode. Meanwhile, for the in-situ-deposited gold-nanoparticle-modified electrode, a stripping peak of gold was observed at 0.24 V, which is much higher than the stripping peak of As(III). As for the electrode modified with in-situ-deposited gold nanoparticles coupled with L-cysteine, the gold stripping peak disappeared, which may be attributed to the binding of L-cysteine on AuNPs, thus preventing the oxidation of gold nanoparticles. When As(III) was added, the stripping peak of As was observed clearly around 0.15 V. The disappearance of the large gold stripping peak made the As(III) peak visible. In our design, the gold nanoparticles can improve the electron transfer of the electrode and L-cysteine can effectively prevent the stripping of gold; thus, the determination of As(III) is made possible.

### 3.4. Optimization of Experimental Parameters

#### 3.4.1. Electrolyte

To investigate the optimal concentration of the components in an electrolyte solution, 10 μg L^−1^ of arsenic was assayed with different concentrations of chloroauric acid (20 µM, 50 µM, 80 µM), L-cysteine (0.2 mM, 0.4 mM, 0.6 mM) and sulfuric acid (0 M, 0.05 M, 0.1 M). A response surface plot (3D) was used to analyze the influences of the three different components. As shown in Figure S2, the slope of the surface plot for chloroauric acid and L-cysteine was the steepest (Figure S2a), and the contours were elliptical, indicating that the interaction between chloroauric acid and L-cysteine was obvious and had the greatest effect on arsenic stripping, followed by sulfuric acid. The peak current values for arsenic initially increased with the increasing concentration of each component (Figure S2); however, as the concentration of each component increased, they created an impedance to the stripping of arsenic and the peak current values thus tended to decrease. Therefore, to achieve the best arsenic stripping effect, the final concentration of each component was 50 μM for chloroauric acid, 0.4 mM for L-cysteine and 50 mM for sulfuric acid. This result is consistent with the results of the response surface ANOVA shown in Figure S2d.

#### 3.4.2. Enrichment Potential and Enrichment Time

**Figure 5:**
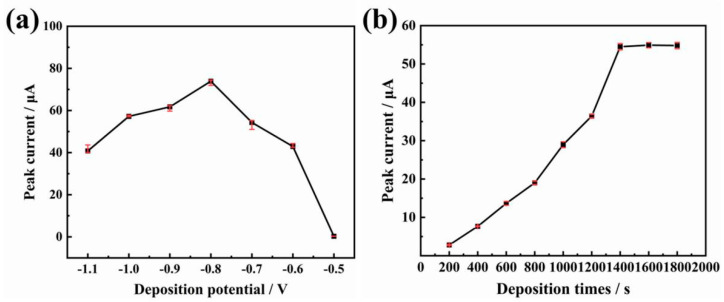
Effect of (**a**) enrichment potential and (**b**) enrichment time on the peak current values of arsenic stripping.

A suitable enrichment potential can effectively improve the determination stability and reduce the interference of other ions. The effect of the enrichment potential on the stripping signal of As(III) was investigated in the range of −0.5 V~−1.1 V. As shown in Figure 5a, when the enrichment potential decreases, the arsenic peak current increases until it reaches the maximum at −0.8 V, and then the peak current starts to decrease. The reduced peak current at less than −0.8 V enrichment potential may be due to the reduction of H^+^ at the low potential, causing a hydrogen evolution phenomenon, which prevents electron transfer on the electrode surface and affects the enrichment efficiency [31]. Therefore, the optimum enrichment potential was set at −0.8 V.

The enrichment time affects the amount of gold nanoparticles deposited on the SPCE. The relationship between the current values of the As(III) stripping peak and the enrichment time varying from 200 s to 2000 s was investigated. As shown in Figure 5b, the As(III) stripping peak current value increased as the enrichment time increased from 200 s to 1400 s and then it reached a plateau. A further increase in the enrichment time did not increase the peak current. Therefore, 1400 s was considered as the optimum enrichment time.

#### 3.4.3. Stirring Speed

During the enrichment process, stirring can cause the ions to distribute evenly in the solution. This results in the better enrichment of gold nanoparticles and As on the electrode to increase the stripping peak current. As shown in Figure S3, the As stripping peak current increased with the increase in the stirring speed and reached the maximum at the stirring speed of 100 rpm. When the stirring rate exceeded 100 rpm, no obvious increase in the peak current was observed. However, the error bar was relatively large. This unstable enrichment on the working electrode surface may be caused by excessively fast stirring. Therefore, 100 rpm was chosen as the optimum stirring speed for the subsequent electrochemical experiments.

### 3.5. Electrochemical Determination of As(III)

**Figure 6:**
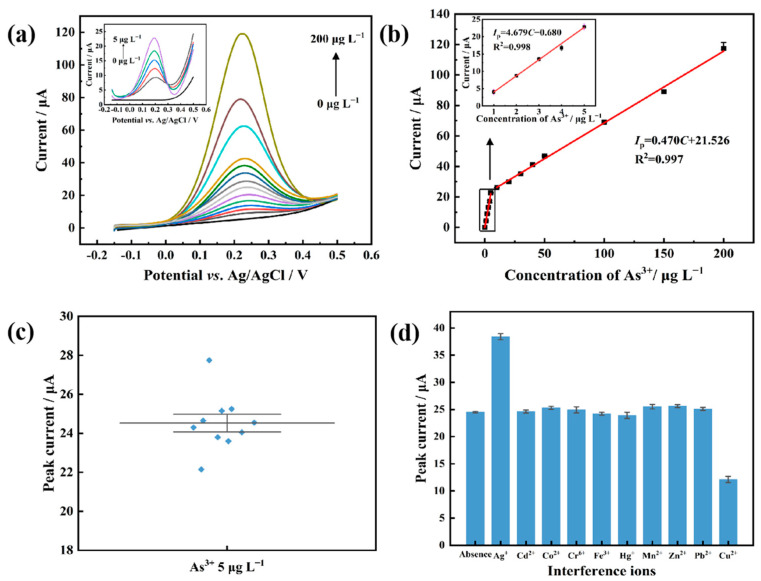
Analytical performance of gold nanoparticle and L-cysteine modified SPCE for As(III) determination. (**a**) The linear anodic stripping voltammetry curves of As(III) (800 µL electrolyte solution: 50 µM chloroauric acid and 0.4 mM L-cysteine in 50 mM H_2_SO_4_. LSASV: scan window −0.15 V to 0.5 V; scan rate is 2 V/s; sampling interval is 0.001 V; rest time is 10 s; deposition time is 1400 s; deposition potential is −0.8 V; stirring speed during deposition was set as 100 rpm). (**b**) Relationship between stripping peak current values and arsenic concentrations. Two linear stand curves were obtained. (**c**) Repeatability of the electrochemical sensor. Ten measurements were conducted in parallel. (**d**) Specificity study of the electrochemical sensor. The concentrations of the interfering ions were 20 times higher than that of As(III). The interfering ion was added to the As(III) solution separately, and the corresponding stripping peak current values of As(III) were compared with that of As(III) solution absent of any in different ions (marked as Absence).

Different concentrations of As(III) (0, 1, 2, 3, 4, 5, 10, 20, 30, 40, 50, 100, 150 and 200 μg L^−1^) were tested under optimal conditions to investigate the linear range and determination limit of the AuNP and L-cysteine co-deposited SPCE. As shown in Figure 6a, with the increase in As(III) concentration, the peak current increased and the peak potential remained unchanged. Results show that the stripping peak current increased with the increasing As(III) concentration, and two linear regression equations were obtained in the ranges of 0~5 μg L^−1^ and 5~200 μg L^−1^, respectively, both with good linear relationships (Figure 6b). The electroactive area of the bare electrode is small and the electron transfer rate is low. When the chloroauric acid begins to reduce to gold nanoparticles on the electrode surface, the electroactive area and electron transfer rate of the electrode’s surface continue to increase and the increases are accelerated as more gold nanoparticles are reduced. When the concentration of arsenic ions in the solution is low, fewer ions need to be reduced. The reduction speed of arsenic on the electrode surface is then fast. Moreover, the gold nanoparticles would also continue to increase the catalytic reaction process, resulting in the high sensitivity of the electrode response. When the concentration of arsenic ions in solution is high, more ions need to be reduced and the speed of arsenic reduction would be slow, resulting in a lower slope. In addition, saturation levels can be reached at higher concentrations. Therefore, different linear relationships were obtained in different concentration ranges [38]. The limit of determination (LOD) was calculated with 3σ/k, where σ is the standard deviation of the peak current values of the blank sample, and k is the slope of the linear curve. The LOD for As(III) determination was determined to be 0.91 ppb. This indicates that the in situ deposition of gold nanoparticles and L-cysteine on the SPCE has a wide linear range and low determination limit for the determination of As(III).

To investigate the repeatability of the proposed electrochemical sensor, ten pieces of SPCE were prepared for the determination of 5 μg L^−1^ As(III) standard solution using the method described in Section 2.3 (Figure 6c). A relative standard deviation (RSD) of 5.3% was calculated based on the ten consecutive measurements, indicating the good repeatability of the As(III) determination methods.

For the specificity examination, Ag(I), Cd(II), Co(II), Cr(VI), Fe(III), Hg(I), Mn(II), Zn(II), Pb(II) and Cu(II), with a concentration 20 times higher than that of As(III), were added into 10 different vials containing 10 μg L^−1^ As(III) solution, respectively. The 10 As(III) solutions with different ions were assayed using the proposed electrochemical sensor. As shown in Figure 6d, compared with the As(III) solution with no other ion, the addition of interfering ions did not change the As(III) stripping peak current significantly, except for Ag(I) and Cu(II), indicating that most of the ions have no effect on the determination of As(III). In consideration of the fact that Ag(I) rarely appears in the actual target samples, the main source of interference in As(III) determination is Cu(II). However, this interference can be masked by adding the appropriate amount of thiourea [39] to the sample. Therefore, the proposed electrochemical sensor can specifically detect As(III) without interference from other ions.

Different electrochemical methods using modified electrodes for the determination of As are summarized in Table 1. In comparison with other methods, our AuNP-L-Cyst/SPCE sensor has a lower determination limit and a wider linear range for the determination of As, with a wider application range.

### 3.6. Recovery Studies

The gold nanoparticle and L-cysteine co-deposited SPCE was used to detect As(III) in tap water and tea samples. Mineral water and tea samples spiked with low, moderate and high levels of As(III) were assayed according to the method described in Section 2.3. As shown in Table 2, the recovery rates ranged from 93.8% to 105.4%, indicating the good accuracy of the electrochemical sensor. In addition, the relative standard deviation (RSD) was lower than 5%, indicating the good repeatability of the sensor. To further validate the feasibility of the constructed electrochemical sensor for As(III) determination, the spiked samples were also determined using ICP-MS. As shown in Table 2, the two methods showed consistent detection results, and the deviation was minimal. The gold nanoparticle and L-cysteine co-deposited SPCE is suitable for rapid and accurate As(III) determination in tap water and tea samples.

The results of the electrochemical and ICP-MS assays were compared by statistical analysis. A *t*-test was used to compare the significance of the difference between the means of the two methods, resulting in *t* (*p* < 0.05) = 3.1. All values for *t*_calculated_ were lower than the *t*_critical_, and the results of the data for the determination of arsenic by the two methods showed no significant difference in the mean values. The *F*-test was used to compare the precision of the data between the two methods, resulting in *F* (*p* < 0.05) = 19.16. The calculated *F* values were lower than *F*_critical_, and this indicates that there is no significant difference in the precision of the data between the two methods.

## 4. Conclusions

In summary, in this study, an in situ deposition method of gold nanoparticles and L-cysteine on an SPCE was prepared for the rapid and sensitive electrochemical determination of As(III). The method does not need complex pre-treatment, and the electrode requires no modification before the electrochemical measurement. The deposition of gold nanoparticles on the electrode and the enrichment of As start simultaneously at the same electrical potential, and the As(III) is then stripped off for determination. The developed sensor showed a wide detection range and good sensitivity, with a determination limit as low as 1 ppb. This method has been verified for the determination of As(III) in tap water and tea samples with good accuracy. The developed method holds great potential for the rapid and on-site determination of As(III). Future studies should focus on the development of portable detection equipment for point-of-care testing.

## Figures and Tables

**Table 1:** Comparison of different electrochemical sensors for As(III) determination.

Electrode	Method	Linear Range (ppb)	LOD (ppb)	Applications	Ref.
PtNPs/SPCE ^1^	CV		5.68	Tap water	[15]
GO/SPCE ^2^	DPV ^3^	0.1~50	0.92	Tap water and urine	[16]
Ag-NP-SPCNFEs ^4^	DPASV	1.9~25.1	1.9	Drinking water	[17]
CNF-CHIT-AuNPs/SPCE ^5^	CV	100~1000	11.4	Tap water	[19]
Pt/GCE ^6^	LSV	4~77	4	Tap water	[20]
GO-MB/Aptamer-AuNPs/SPCE	DPV	0.4~1000	0.2	Shellfish	[21]
AuNPs/SPCE	SWV ^7^		16.73	Apple juice	[22]
SiNPs/SPCE ^8^	LSASV	5~30	6.2	Tap water	[23]
AF-AuNPs/SPCE ^9^	CV	6~200	6	River water	[24]
AuNPs-L-Cyst/SPCE	LSV	1~200	0.91	Tap water and tea leaf	This work

^1^ PtNPs: platinum nanoparticles; ^2^ GO: graphene oxide; ^3^ DPV: differential pulse voltammetry; ^4^ Ag-NP-SPCNFEs: carbon-nanofiber-based SPCE modified with silver nanoparticles; ^5^ CNF-CHIT-AuNPs: gold-nanoparticle-decorated carbon nanofiber–chitosan; ^6^ GCE: glassy carbon electrode; ^7^ SWV: square wave voltammetry; ^8^ SiNPs: silica nanoparticles; ^9^ AF-AuNPs: *Alcaligenes faecalis* immobilized on a gold nanoparticle.

**Table 2:** Results of As(III) determination in tap water and tea samples.

Sample	Added (μg L^−1^)	Found (μg L^−1^)	Recovery/%	RSD/%	Found by ICP-MA (μg L^−1^)	|Error|	*t* ^1^	*F* ^2^
Tap water 1	0	0						
	10	12.3	105.4	3.18	11.9	3.4%	0.13	0.35
	50	48.2	98.8	2.15	49.4	2.4%	0	0.19
Tap water 2	0	0						
	10	10.6	101.2	4.02	10.2	3.8%	0.56	0.03
	50	51.4	99.3	1.99	50.8	1.1%	0	0.12
Tea leaf 1	0	0			2.3			
	10	11.3	97.6	4.57	11.9	5.0%	0.09	0.23
	50	52.6	102.6	3.06	52.7	0.18%	0.76	0.29
Tea leaf 2	0	0						
	10	10.8	98.1	4.88	10.5	2.8%	0.94	0.10
	50	51.1	97.7	3.71	51.7	1.2%	0	0.44
Tea leaf 3	0	0						
	10	8.8	96.2	1.32	9.1	3.2%	0.05	0.08
	50	47.2	93.8	2.94	48.6	2.9%	0	0.43

^1^ *t*_critical_ = 3.1; ^2^ *F*_critical_ = 19.16.

## Data Availability

All data are contained within the article or the Supplementary Materials.

## Supplementary Materials

The following supporting information can be downloaded at: https://www.mdpi.com/article/10.3390/bios13010130/s1, Figure S1: Illustration of the self-made stirring device. (a) SPCE and matched connector. (b) The constitution of the stirring device. Figure S2: Influence of the concentration ratio of the components in the mixed stock solution on the stripping of As(III). (a) Chloroauric acids and L-cystine. (b) Chloroauric acids and H_2_SO_4_. (c) L-cystine and H_2_SO_4_. (d) Analysis of variance (ANOVA) for response surface quadratic model. Figure S3: Effect of stirring speed on the peak current values of arsenic stripping.
