# Supplementary material for: In Situ Deposition of Gold Nanoparticles and L-Cysteine on Screen-Printed Carbon Electrode for Rapid Electrochemical Determination of As(III) in Water and Tea
Source: Biosensors (Basel). 2023 Jan 12;13(1):130. doi: 10.3390/bios13010130 (PMC9856477; doi:10.3390/bios13010130)
Supplement: Supplementary file 1 [file biosensors-13-00130-s001.zip › biosensors-2137478-supplementary.pdf]

Supplementary Materials

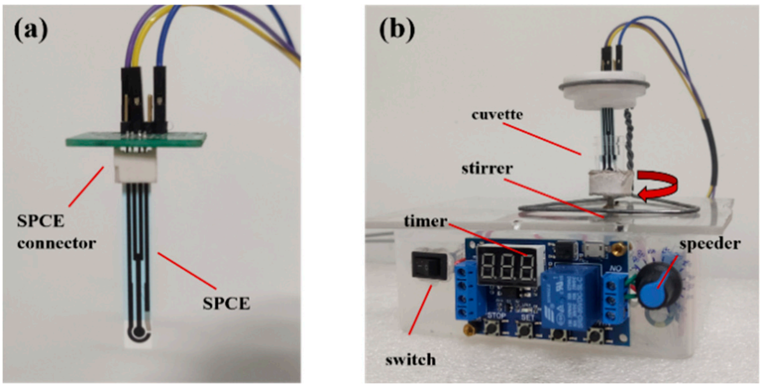

Figure S1. Illustration of the self-made stirring device.(a) a screen-printed electrode and a matched connector. (b) a home made stirring device.

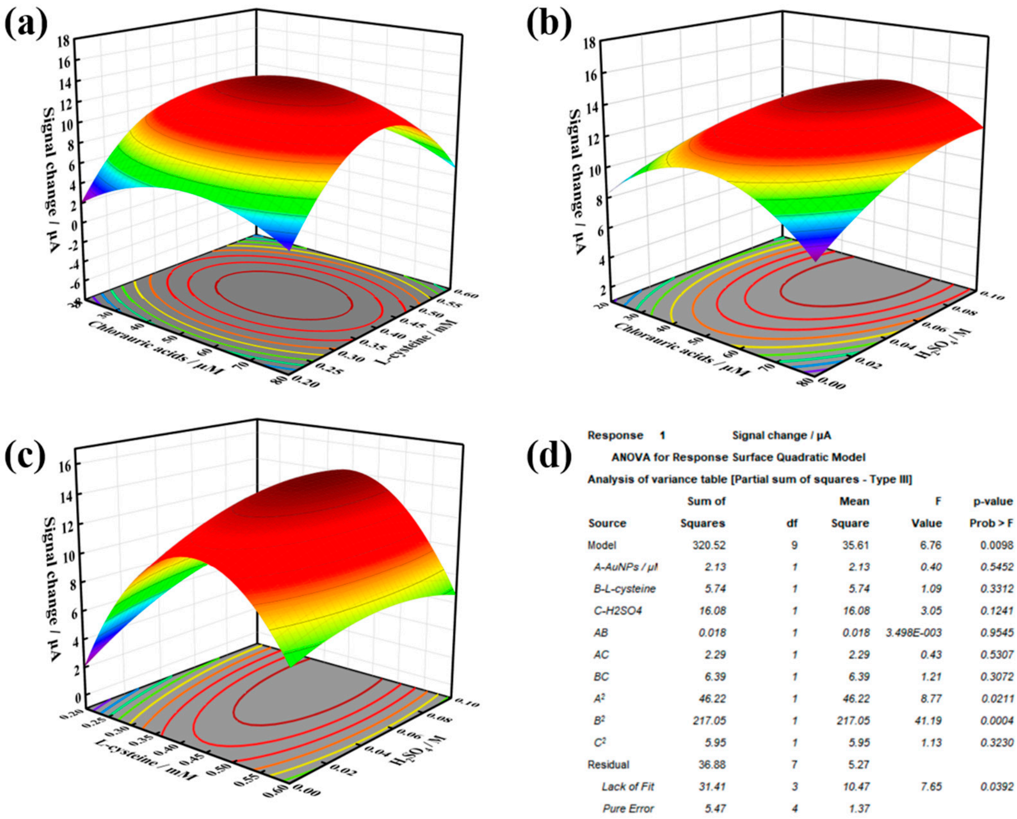

(d)

Response 1 Signal change / μA

ANOVA for Response Surface Quadratic Model

Analysis of variance table [Partial sum of squares - Type III]

Source	Sum of Squares	df	Mean Square	F Value	p-value Prob > F	
Model	320.52	9	35.61	6.76	0.0098	significant
A-AuNPs / μl	*2.13*	*1*	*2.13*	*0.40*	*0.5452*	
B-L-cysteine	*5.74*	*1*	*5.74*	*1.09*	*0.3312*	
C-H2SO4	*16.08*	*1*	*16.08*	*3.05*	*0.1241*	
AB	*0.018*	*1*	*0.018*	*3.498E-003*	*0.9545*	
AC	*2.29*	*1*	*2.29*	*0.43*	*0.5307*	
BC	*6.39*	*1*	*6.39*	*1.21*	*0.3072*	
A^2	*46.22*	*1*	*46.22*	*8.77*	*0.0211*	
B^2	*217.05*	*1*	*217.05*	*41.19*	*0.0004*	
C^2	*5.95*	*1*	*5.95*	*1.13*	*0.3230*	
Residual	36.88	7	5.27			
Lack of Fit	*31.41*	*3*	*10.47*	*7.65*	*0.0392*	*significant*
Pure Error	*5.47*	*4*	*1.37*			
Cor Total	357.40	16				

Figure S2. Influence of the concentration ratio of the components in the mixed stock solution on the stripping of As(III). (a) chloroauric acids and L-cystine (b) chloroauric acids and H_2SO_4 (c) L-cystine and H_2SO_4 (d) ANOVA for Response Surface Quadratic Model.

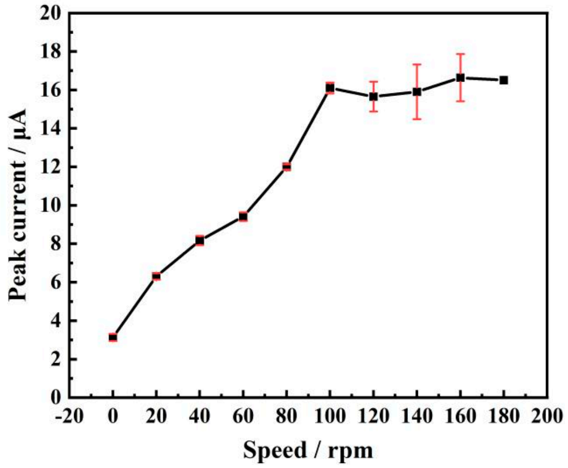

Figure S3. Effect of stirring speed on the peak current values of arsenic solution.
